# Thoracic Imaging Utilization on Super Bowl Sundays: A Retrospective Analysis From a Large Academic Medical Center

**DOI:** 10.7759/cureus.108050

**Published:** 2026-04-30

**Authors:** Shilpa Chandran, Bilal El Kaddouri, Isha Gujrathi, Seyedeh Panid Madani, Domenic Mongillo, Sara J Schiller, Maria F Barile, Alexander A Bankier

**Affiliations:** 1 Radiology, UMass Chan Medical School, Worcester, USA

**Keywords:** chest radiography, ct (computed tomography) imaging, health services utilization, thoracic imaging, turnaround time (tat)

## Abstract

Purpose

Super Bowl Sundays (SBSs) have been linked to shifts in social behavior and health-related events. Understanding its impact on thoracic imaging utilization can guide radiology staffing and resource planning during major public events. This single-center study aimed to evaluate thoracic imaging patterns on SBSs within our healthcare network.

Materials and methods

We retrospectively analyzed data from the two central tertiary-care campuses of our healthcare network for SBSs in 2022, 2023, and 2024, using the adjacent Sundays and Saturdays as temporal controls. Two time windows were evaluated: entire days (00:00-23:59) and television (TV) prime time (17:00-22:59). Data included patient demographics, imaging type, and timing details. Poisson and robust regression models were used to assess differences in patient and exam volumes per hour and turnaround times (TATs), adjusting for year and time of day.

Results

A total of 3,894 thoracic imaging examinations performed in 3,148 patients were analyzed. On SBSs, significantly fewer patients were seen compared to control Sundays (6.7±0.8 vs 7.2±0.7; *P*=0.019), and fewer chest radiographs were performed (7.0±0.8 vs 7.8±0.8; *P*=0.019). During TV prime time, the significant drop was more pronounced in radiograph volumes (6.1±0.5 vs 8.0±0.4; *P*=0.012), alongside a significant reduction in TATs (37±12 vs 48±13 min; *P*=0.024). No such differences were seen on control Saturdays. No significant differences were seen in CT volumes or TATs on SBSs.

Conclusion

Thoracic imaging utilization, especially chest radiographs, decreased on SBSs, driven by fewer patient visits, particularly during prime time. This corresponded with shorter TATs, despite a trend toward more exams per patient.

## Introduction

The relation between Super Bowl Sundays (SBSs) and various medically relevant events has been previously studied [1-7]. These medically relevant events and occurrences include the frequency of emergency room visits [8-10], the incidence of cardiovascular events [1,2,11], the rates of domestic violence [12,13], and the number of traffic accidents [5]. If consequences of these events and occurrences entail a hospital visit, they will almost inevitably trigger utilization of diagnostic imaging and, notably, of thoracic imaging. It, therefore, appears reasonable to assume that there could be a potential association between SBSs and thoracic imaging utilization. However, no previous study has tested this assumption.

If confirmed, potential relations between SBSs and thoracic imaging utilization could help optimize planning for thoracic radiology services on this day, which might be practically relevant in times of understaffing and increased clinical workloads [14-18]. It could also help to increase preparedness for other upcoming major sports events, such as the FIFA World Cup, to be held in the United States in 2026 [19]. The purpose of our study, therefore, was to analyze the relation between SBSs and thoracic imaging utilization in our healthcare network and to identify potential utilization patterns related to this event.

## Materials and methods

Study setting

This single-center retrospective study was conducted at two central tertiary-care campuses of a large academic medical center on the US East Coast. This study was reviewed by the Institutional Review Board and was deemed exempt, as it involved retrospective analysis of imaging utilization data without collection of patient identifiers (H#00020422).

Study period, days, and time windows

Our study included the SBSs of 2022, 2023, and 2024. We did not extend the study period prior to 2022 to avoid potential confounding by the margins of the coronavirus pandemic and the related substantially decreased outpatient hospital visits [20-23].

We compared data from SBSs to data from the Sundays immediately preceding and following SBSs. To provide an internal control, we also compared data from the Saturdays before SBS to the Saturdays immediately preceding and following SBSs. Within these predefined days, two distinct time windows were analyzed: 1) entire days, ranging from 00:00 to 23:59 hours, and 2) SBS television (TV) prime time, ranging from 17:00 to 22:59 hours. This approach is identical or strongly similar to previous studies investigating the impact of SBS on various aspects of medicine and public health [10,24]. Like several of these previous studies, we assessed inclement weather at our study site as a potential confounder for all calendar weekends covered by this study [25].

Inclusion and exclusion criteria

**Figure 1:**
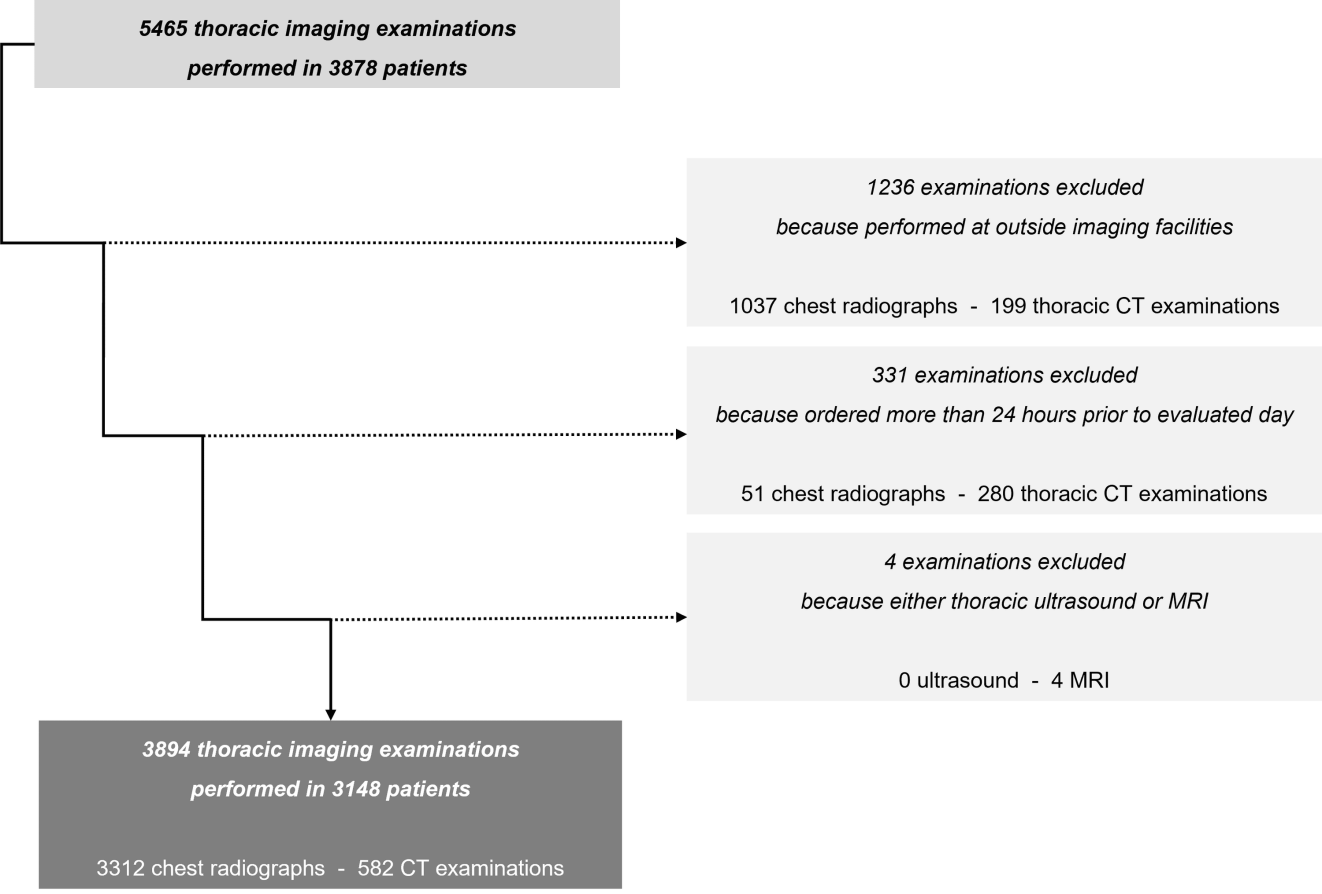
Study Flow Diagram of Patient and Imaging Examination The figure shows the study flow diagram with the number of patients and examinations originally included and then incrementally excluded, as defined by the inclusion and exclusion criteria of the study.

We only included patient and imaging examinations from our two central tertiary care campuses to ensure data homogeneity resulting from local standardized clinical and imaging workflows. Patients and examinations from other network facilities were excluded. We included all chest radiographs as well as all thoracic CT examinations. All other thoracic imaging examinations, such as MRI and ultrasound examinations, were excluded. We only included imaging studies ordered 24 hours or less before completion within the days and time windows analyzed in this study, to avoid potential confounding by routine studies ordered longer in advance and coincidentally scheduled on the SBS. Imaging studies ordered more than 24 hours before completion within the days and time windows analyzed in this study were excluded. Inclusion and exclusion criteria were uniformly applied to all days and time windows analyzed in this study. The numbers resulting from the inclusion and exclusion process are shown in the study flow diagram [26] in Figure 1.

Data analysis

All data analyzed in this study were retrieved from our institution’s information system database (Epic Hyperspace (Version: November 2024, Epic Systems Corporation, Verona, WI), running on Hyperdrive (Version 100.2504.4.0) and HSWeb (Version 11.2.0.8). Data was extracted via the integrated SlicerDicer tool.

For all days and time windows analyzed in this study, and for all patients, we retrieved patient status at the time of imaging, categorized as inpatient (hospitalized patient), outpatient (scheduled appointment), or emergency department patient, as well as age and sex. For the patient-category, outpatients and emergency department patients were grouped together, as the study focused on weekend periods during which routine scheduled outpatient activity was limited. Moreover, for all thoracic imaging examinations performed during the days and time windows analyzed in this study, we retrieved the examination type (chest radiograph or thoracic CT), as well as the time of order and examination completion. For each predefined day and each hour of the day, the number of patients and thoracic imaging examinations was averaged over the three years included in this study. Turnaround time (TAT) was defined in this study as order-to-completion time, corresponding to the time elapsed between the order and the completion of the examination.

The numbers of patients and of examinations per hour were modeled using Poisson regression based on the underlying raw hourly count data, with adjustment for year and hour of the day. Overdispersion was assessed and was not found to substantially affect the Poisson models. This approach is suited for modeling counted data for estimation of rates and effect of covariates on event counts [27]. Patients with more than one thoracic imaging examination were only counted once, but if one patient had more than one examination all of these examinations were counted. TATs were analyzed using robust regression models with adjustment for year and hour of the day. Robust regression is designed to provide reliable results, particularly when the data includes outliers or shows high variability and was therefore well-suited for the nature of our clinical data [28]. Pearson chi-squared tests were used to analyze categorical variables. All statistical analyses were performed using R-software (version 4.4.2, R Foundation for Statistical Computing, Vienna, Austria). The normality of distributions was assessed using the Shapiro-Wilk test [29]. Normally distributed data were expressed as means±standard deviation. Non-normally distributed data was expressed as medians with their 25% and 75% quartiles. Proportions were expressed as fractions of whole numbers, with their corresponding percentage. All statistical tests were two-sided, and P ≤.050 was set as threshold for statistical significance. No formal adjustment for multiple comparisons was applied, as the analyses were predefined and intended to assess a limited number of prespecified outcomes and time windows.

## Results

The dates and game locations, as well as game times and local weather at the study site, are shown in Table 1.

**Table 1:** Super Bowl Dates, Locations, Kick-off Times, and Local Weather at Study Site The table shows the year, weekend category, dates (game date in bold), game location, kick-off time (local at study site), and local weather at the study site for the three years included in this study. SBS: Super Bowl Sunday

Year	Weekend category	Weekend dates	Game location	Kick-off time (local at the study site)	Local weather at the study site
2022	Weekend preceding SBS	Sat 02/05, Sun 02/06	—	—	Sunny and cloudy; 11°F to 24°F
2022	SBS weekend	Sat 02/12, Sun 02/13	Inglewood, CA	6:30 PM EST	Sunny and cloudy; light snow on Sunday; 18°F to 52°F
2022	Weekend after SBS	Sat 02/19, Sun 02/20	—	—	Light snow below 1 inch; otherwise mostly sunny; 32°F to 54°F
2023	Weekend preceding SBS	Sat 02/04, Sun 02/05	—	—	Mostly sunny; 15°F to 47°F
2023	SBS weekend	Sat 02/11, Sun 02/12	Glendale, AZ	6:30 PM EST	Sunny and cloudy; 39°F to 45°F
2023	Weekend after SBS	Sat 02/18, Sun 02/19	—	—	Mostly sunny; 36°F to 55°F
2024	Weekend preceding SBS	Sat 02/03, Sun 02/04	—	—	Mostly sunny; 36°F to 38°F
2024	SBS weekend	Sat 02/10, Sun 02/11	Las Vegas, NV	6:30 PM EST	Sunny and cloudy; 45°F to 55°F
2024	Weekend after SBS	Sat 02/17, Sun 02/18	—	—	Cloudy; 30°F to 32°F

Overall, our study included 3894 thoracic imaging examinations performed in 3148 patients. Examinations were excluded if they were performed at outside imaging facilities (n=1236), ordered more than 24 hours prior to the evaluated day (n=331), or were thoracic ultrasound or MRI studies (n=4). Table 2 summarizes the average number and category of patients analyzed in this study.

**Table 2:** Patient Distribution on Super Bowl and Control Days The table shows average patient numbers and patient categories for the entire day and TV prime time (averages were calculated over three Super Bowl weekends and six control weekends). P-values were calculated using Pearson's chi-squared test. SBS: Super Bowl Sunday; ER: Emergency Room; TV: Television

Time window	Patient category	SBS	Control Sundays	Saturday preceding SBS	Control Saturdays	χ²	P-value
Entire day (00:00–23:59)	In-patients	75/160 (46.9%)	76/174 (43.7%)	87/182 (47.8%)	86/180 (47.8%)	3.9	0.27
Entire day (00:00–23:59)	Out- and ER-patients	85/160 (53.1%)	98/174 (56.3%)	95/182 (52.2%)	94/180 (52.2%)	—	—
TV prime time (17:00–22:59)	In-patients	14/40 (35.0%)	20/51 (39.2%)	25/53 (47.2%)	22/53 (41.5%)	5.4	0.14
TV prime time (17:00–22:59)	Out- and ER-patients	26/40 (65.0%)	31/51 (60.8%)	28/53 (52.8%)	31/53 (58.5%)	—	—

Patient age distribution did not differ between the Super Bowl and control periods for the full day (χ² = 14.2, P = 0.70) or during TV prime time (χ² = 16.3, P = 0.59). Similarly, sex distribution did not differ between periods for the full day (χ² = 1.3, P = 0.74) or during TV prime time (χ² = 0.7, P = 0.88).

**Figure 2:**
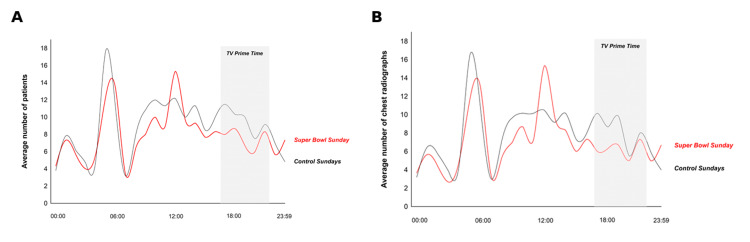
Comparison of Hourly Patient and Chest Radiograph Volume on Super Bowl and Control Sundays The figure shows the average number of patients (A) and average number of chest radiographs (B) on Super Bowl Sunday (red line) and on control Sundays (black line). Both patient numbers and numbers of chest radiographs were lower on Super Bowl Sundays than on control Sundays, notably during TV prime time, with a peak around noon.

Figure 2 shows comparison of hourly patient and chest radiograph volume on Super Bowl and control Sundays.

Analysis of entire days (hours 00:00 to 23:59)

Full-day analyses are summarized in Table 3. On the Saturday preceding the SBS, patient volume, chest radiograph volume, and turnaround times did not differ significantly from control Saturdays, whereas CT examination volume was slightly higher. On the SBS, patient volume and chest radiograph volume were lower than on control Sundays, whereas chest radiograph turnaround time, CT examination volume, and CT examination turnaround time did not differ significantly.

**Table 3:** Full-Day Analyses (Hours 00:00 to 23:59) Values are presented as mean ± standard deviation. P-values for patient and examination counts were derived from Poisson regression models adjusted for year and hour of day. P-values for TAT were derived from robust regression models adjusted for year and hour of day. SBS: Super Bowl Sunday; TAT: turnaround time.

Comparison	Patients per hour	P-value	Chest radiographs per hour	P-value	Chest radiograph TAT, min	P-value	CT examinations per hour	P-value	CT examination TAT, min	P-value
Saturday preceding SBS vs control Saturdays	7.6±0.7 vs 7.5±0.7	0.67	7.7±0.8 vs 7.8±0.8	0.78	153±13 vs 159±19	0.84	1.6±0.2 vs 1.3±0.2	0.047	224±22 vs 217±48	0.38
SBS vs control Sundays	6.7±0.8 vs 7.2±0.7	0.02	7.0±0.8 vs 7.8±0.8	0.02	143±59 vs 128±18	0.71	1.3±0.3 vs 1.3±0.2	0.67	191±20 vs 177±44	0.46

Analysis of TV prime time (hours 17:00 to 22:59)

TV-prime-time analyses are summarized in Table 4. During TV prime time on the Saturday preceding the SBS, no significant differences were observed relative to control Saturdays. In contrast, during TV prime time on SBS, patient volume and chest radiograph volume were lower than on control Sundays, and chest radiograph turnaround time was shorter. No significant differences were observed in CT examination volume or CT examination turnaround time during this time window.

**Table 4:** TV-Prime-Time Analyses (Hours 17:00 to 22:59) Values are presented as mean ± standard deviation. P-values for patient and examination counts were derived from Poisson regression models adjusted for year and hour of day. P-values for TAT were derived from robust regression models adjusted for year and hour of day. SBS: Super Bowl Sunday; TAT: turnaround time.

Comparison	Patients per hour	P-value	Chest radiographs per hour	P-value	Chest radiograph TAT, min	P-value	CT examinations per hour	P-value	CT examination TAT, min	P-value
Saturday preceding SBS vs control Saturdays	8.8±2.2 vs 8.8±1.2	0.53	7.9±1.8 vs 8.4±1.5	0.52	68±11 vs 62±19	0.44	1.8±0.8 vs 1.6±0.6	0.21	239±49 vs 205±69	0.16
SBS vs control Sundays	6.6±0.9 vs 8.5±0.2	0.02	6.1±0.5 vs 8.0±0.4	0.01	37±12 vs 48±13	0.02	1.6±1.0 vs 1.6±0.2	0.94	232±115 vs 114±27	0.08

## Discussion

Our study shows that on SBSs, both over the entire day and during TV prime time, significantly fewer chest radiographs were performed in our department than on control Sundays (6.1±0.5 vs 8.0±0.4; P=0.01). Simultaneously, on SBSs, and again both over the entire day and during TV prime time, significantly fewer patients underwent thoracic imaging in our department than on control Sundays (6.6±0.9 vs. 8.5±0.2; P=0.02). This suggests that the lower number of chest radiographs performed on SBSs is related to the “Super Bowl” and likely driven by the lower number of patients presenting to our department on SBSs. This is supported by the absence of similar differences in both the number of patients (8.8±2.2 vs 8.8±1.2; P=0.53) seen in our department and of chest radiographs (7.9±1.8 vs 8.4±1.5; P=0.52) performed on Saturdays.

Previous studies found relations between SBS-related medical events and occurrences, and age and sex of patients [1]. No such relations were seen in our study. Indeed, there were no significant differences in age and sex of the patients presenting to our department on SBSs and control Sundays, as well as on the Saturday preceding the SBS and control Saturdays. This absence of significant differences should be interpreted in the context of average patient and chest radiograph numbers that were of similar magnitude to those previously reported [14-18].

A practical aspect of the decreased chest radiograph utilization on SBSs was a decrease in TATs. For unexplained reasons, this decrease reached statistical significance for the TV prime time hours only but did not translate over the entire day. Previous studies have suggested staffing changes to respond to decreased clinical workload on SBSs [9]. We believe that the observations from this study are too limited in time to justify such changes, at least for the radiology department at our institution.

Our study found no significant differences in the number of CT examinations performed on SBSs and on control Sundays, which was consistent for both entire Sundays and TV primetime. Likewise, no significant differences in TATs for CT examinations were seen for the various days and time windows. This can be explained by the fact that CT examinations are procedurally and technically more complex than chest radiographs and are organized along a more structured process, including patient transport and preparation, as well as image processing and reconstruction. Therefore, they could be less prone than chest radiographs to a limited event-dependent change in patient flow.

Two other observations from this study deserve discussion. On the Saturday preceding the SBS, CT examination volume was higher than on control Saturdays, whereas patient volume did not differ significantly. Although speculative, this pattern, together with the lower patient volume observed on SBS itself, may suggest that some patients presented before rather than during SBSs. This could indicate that, if planning is possible, a subgroup of patients preferred to present to our department on the day before the game rather than on SBS itself, which could have triggered a higher number of CT examinations on this particular Saturday. A similar attitude could have caused the peak of patient visits and subsequent chest radiographs around noontime of SBS. A subgroup of patients might have chosen to get their hospital visit done earlier on SBS, to not miss the game later in the day. Furthermore, on SBSs, the examination-per-patient ratio was slightly higher than on control Sundays and Saturdays. As sicker patients could require more imaging examinations, a higher examination-per-patient ratio could hypothetically serve as a surrogate metric for sicker patients. If accurate, this could indicate that on SBSs, fewer but sicker patients were seen in our department.

Our study has several limitations. First, our study was conducted at a single academic medical center. We, therefore, do not know how generalizable our findings are to other medical centers, both in our State and in the US at large. Second, our study covered only three years of SBSs, and we did not extend it to earlier years, to avoid potential confounding by outbreaks of the COVID-19 pandemic and the resulting changes in outpatient hospital visits [20-23]. Our study, therefore, does not provide a long-term longitudinal perspective on the potential relation between SBS and thoracic imaging, and the small number of SBSs may have limited our ability to detect more subtle differences. Moreover, we did not include direct measures of patient acuity, nor did we assess possible differences in staffing or workflow across the study periods. In addition, we did not analyze total hospital or emergency department census data and therefore cannot determine whether the observed differences reflected broader changes in healthcare utilization beyond thoracic imaging. Finally, relation is not causation. Although we have identified relations between SBS and thoracic imaging utilization, we cannot determine to what extent SBSs did indeed cause the observed changes, as we might be missing unknown factors of influence.

## Conclusions

In conclusion, SBSs were associated with a measurable short-term change in thoracic imaging utilization at our institution, particularly for chest radiographs. The observed pattern suggests that major televised events may influence patient presentation and, in turn, radiology workflow, particularly during evening game hours. However, these findings reflect association rather than causation and derive from a single academic medical center across three SBSs. They should therefore be interpreted cautiously. Further studies are needed to determine whether similar patterns are present across other institutions and radiology settings.
